# Pro-Inflammatory Signature in Decidua of Recurrent Pregnancy Loss Regardless of Embryonic Chromosomal Abnormalities

**DOI:** 10.3389/fimmu.2021.772729

**Published:** 2021-12-08

**Authors:** Zaigui Wu, Miaomiao Wang, Guanmian Liang, Pengzhen Jin, Peng Wang, Yuqing Xu, Yeqing Qian, Xiuxiu Jiang, Junbin Qian, Minyue Dong

**Affiliations:** ^1^ Women’s Hospital, Zhejiang University School of Medicine, Hangzhou, China; ^2^ Cancer Hospital of University of Chinese Academy of Sciences, Zhejiang Cancer Hospital, Hangzhou, China; ^3^ Key Laboratory of Women’s Reproductive Health of Zhejiang Province, Hangzhou, China; ^4^ Key Laboratory of Reproductive Genetics, Ministry of Education, Zhejiang University, Hangzhou, China

**Keywords:** recurrent pregnancy loss, decidua, CyTOF, embryonic chromosomal aberrations, maternal immune tolerance

## Abstract

Recurrent pregnancy loss (RPL), especially the unexplained RPL, is associated with the disruption of maternal immune tolerance. However, little is known about the immune status at the decidua of RPL with embryonic chromosomal aberrations. Herein, mass cytometry (CyTOF) was used to interrogate the immune atlas at the decidua which was obtained from 15 RPL women—six with normal chromosome and nine with chromosomal aberrations—and five controls. The total frequency of CCR2^−^CD11c^high^ macrophages increased, while CD39^high^ NK cells and CCR2^−^CD11c^low^ macrophages decrease significantly in RPL when RPLs were stratified, compared with controls. Pro-inflammatory subsets of CD11c^high^ macrophages increased, while less pro-inflammatory or suppressive subsets decreased statistically in RPL decidua whenever RPLs were stratified or not. However, CD11c^high^ NK and CD161^high^CD8^+^ T cells increased only in RPL with normal chromosome, while the inactivated and naive CD8^+^/CD4^+^ T cells were enriched only in RPL with chromosomal aberrations. A pro-inflammatory signature is observed in RPL decidua; however, differences exist between RPL with and without chromosomal abnormalities.

## 1 Introduction

Recurrent pregnancy loss (RPL) is defined as two or more consecutive pregnancy losses before the 20th week of gestation and occurs in about 1%–3% of reproductive women (1). With the number of miscarriages increased, the recurrent risk of pregnancy loss raises quickly from about 11% at one loss to 40% after three or more losses (2). Though possible causes including uterine malformations, maternal infection, endocrine disorders, and genetic abnormalities have been identified, only maternal age and the number of prior pregnancy losses are considered as prognostic factors for majority of RPL women (1). No obvious cause can be found in more than 50% of RPL even after comprehensive investigation, and they are called as unexplained RPL (3).

It has been proposed that RPL, especially the unexplained RPL, arises from the disruption of maternal immune homeostasis (4). Innate lymphoid cells (ILCs), myeloid cells, T cells and B cells contribute to the maintenance of tolerance in pregnancy (5–7). ILCs, including NK cells and non-NK ILCs, are the most abundant immune cells in a pregnant uterus. Decidual NK cells (dNKs) possess a CD56^bright^CD16^−^ phenotype and account for 50%–70% of leukocytes in the first trimester. They not only modulate trophoblast invasion by producing IL-8 and IL-10 but also initiate artery remodeling through secreting vascular endothelial growth factor (VEGF) and arginase-1 (5, 6). dNK plays an important role in extravillous trophoblast (EVT) cells acquiring invasive phenotype and invasive EVT gaining endothelial-like properties (8). Decidual non-NK ILCs are mainly ILC3 and participate in tissue remodeling by expressing IL-22 and IL-8 or inducing decidual stromal cell (DSC) to express adhesion molecules (9).

Myeloid cells, mainly including macrophages and dendritic cells (DCs), remain relatively high and constant during gestation. Decidual macrophages play an essential role in inducing maternal immune tolerance, modulating vascular modeling and trophoblast invasion (10). DCs are the most efficient type of antigen-presenting cells and can drive naive CD4^+^ T-cell polarization to a Th2 phenotype (11).

Decidual CD4^+^/CD8^+^ T cells are associated with recognizing fetal antigens and regulating decidual immune microenvironment, and activated CD4^+^/CD8^+^ T cells and Foxp3^+^ Tregs are essential in mediating immunological tolerance to the fetus (12). B cells, particularly Breg cells, can induce maternal tolerance by producing IL-10 or the CD39/CD73 pathway (13). Immune dysregulations from each type of cells are associated with RPL.

In unexplained RPL women aged less than 35 years of age, the proportion of blastocyst aneuploidy increased, but the clinical miscarriage rate is higher when they have euploid embryos transferred by preimplantation genetic testing (PGT) than that in women with PGT just for monogenic defects (14). In women over 35 years old aneuploidy rates neither in embryo nor in blastocysts are not significantly different between sporadic abortion and RPL or idiopathic RPL (14, 15). These findings indicate that embryonic chromosomal abnormalities are not the only cause for RPL and may occur no matter how old women are.

Trisomy 21 (T21) is one of the most common chromosomal abnormalities in RPL and is also the most widespread survival autosomal aneuploidy. The incidence of leukemia is approximately 50-fold higher in children with T21 compared with normal children in the first 5 years of life (16), while adults with T21 are predisposed to autoimmune diseases and global immune dysregulations (17). These imply that the extra copy of chromosome 21 in embryo may induce immune variations. Thus, we hypothesized that immune abnormalities might exist in RPL with embryonic chromosomal aberrations.

Single-cell technologies such as CyTOF and single-cell RNA sequencing (scRNA-seq) have been used to explore the immune-related physiological mechanism in early or peripartum decidual tissue and endometrium across the menstrual cycle (5, 18, 19). Though a few studies using scRNA-seq have demonstrated a systematic pro-inflammatory feature in RPL with normal embryonic chromosome (20–23), the global immune landscape of RPL, especially those with embryonic chromosomal aberrations, is still unknown.

Herein, we harnessed the power of CyTOF and tried to reveal the immune phenotypic characteristics of RPL with or without embryonic chromosomal aberrations. We observed a pro-inflammatory signature in RPL whether the embryonic chromosome is normal or not, and we also noticed differences between RPL with and without chromosomal abnormalities.

## 2 Materials and Methods

### 2.1 Subjects

Fifteen RPL women, six with normal chromosome and nine with chromosome aberrations, and five controls were enrolled from May 2020 to October 2020 at Women’s Hospital, Zhejiang University School of Medicine. The baseline characteristics were summarized in **Table 1**. RPLs were diagnosed according to the criteria of ESHRE 2017 (1). RPL women with autoimmune disorders such as systemic lupus erythematosus, antiphospholipid antibody syndrome, and those prescribed with immunosuppressor, aspirin, or low molecular heparin were excluded from this study. Five women undergoing artificial abortions with a similar gestation served as controls. They should not have the above autoimmune disorders and prior miscarriages and stillbirth. The protocol was approved by the ethics committee of the hospital (IRB-20200126-R) and informed consents were obtained from all participants.

**Table 1:** Main characteristics of the subjects.

Case	Age (years)	GmPn	Number of SA	GD	MC/MP (days)	Ultrasound presentation	Karyotype/methods
BL9	21	G2P0	2	54	30/4–5	GS 3.6 cm	NK/CNV-seq
BL35	30	G2P0	2	56	30/5	CRL 0.5 cm	NK/CMV
BL34	27	G3P0	3	62	37/7	CRL 0.3 cm	T16/CMV
BL33	30	G5P0	2	71	30–50/7	GS 3.1 cm	NK/CNV-seq
BL3	41	G4P1	3	71	28/5	CRL 3.1 cm	T21/CMV
BL27	33	G3P0	3	61	Unknown	CRL 0.14 cm	NK/CMV
BL22	26	G2P0	2	71	20–60/7	CRL 0.3 cm	NK/CMV
BL21	29	G2P0	2	68	37/7	GS 2.5 cm	4P/CMV
BL20	40	G2P0	2	67	Unknown	CRL 1.0 cm	T21/CMV
BL2	40	G4P1	3	72	28–30/5	CRL 2.7 cm	T21/CMV
BL19	30	G2P0	2	70	30/5–6	CRL 0.4 cm	T22/CMV
BL17	36	G3P1	2	71	28–29/5–6	CRL 0.5 cm	T16/CMV
BL16	29	G2P0	2	61	25/4	CRL 1.4 cm	NK/CMV
BL13	27	G3P0	3	47	28–32/5	GS 1.0 cm	T22/CMV
BL09	24	G3P0	3	75	30/4–5	CRL 2.1 cm	T21/CMV
NC4	24	G2P0	0	52	30/5	CRL 1.0 cm	NK/CNV-seq
NC7	36	G3P1	0	61	60/4–5	CRL 1.2 cm	NK/CNV-seq
NC10	38	G3P1	0	64	45–60/7	CRL 1.8 cm	NK/CNV-seq
NC11	24	G1P0	0	70	28/5	CRL 3.5 cm	NK/CNV-seq
NC13	24	G2P0	0	67	30/5	Unknown	NK/CNV-seq

GD, gestational days; SA, spontaneous abortion; MC, menstrual cycle; MP, menstrual period; GS, gestational sac; CRL, crown–rump length; CMA, chromosomal microarray analysis; CNV-seq, copy number variation sequencing; NK, normal karyotype; T21, trisomy 21; T16, trisomy 16; T22, trisomy 22.

### 2.2 Villus Separation and Embryonic Chromosome Analyses

Villus was obtained and separated as previously described (24) and was stored at −80°C before use. Chromosomal microarray analysis (CMA) or copy number variation sequencing (CNV-seq) was used to analyze the karyotypes of villus.

### 2.3 Mass Cytometry

#### 2.3.1 Decidual Tissue Single-Cell Dissociation and Sample Preparation

Decidual tissues were washed with saline, collected in ice-cold MACS Tissue storage solution (catalog no. 130-100-008, Miltenyi, Germany) and dissociated into single cells. CyTOF analyses were performed by PLT Tech Inc. (Hangzhou, China). In brief, cells were stained 5 min with 5 µM 194Pt cisplatin (Fluidigm) for viability in phosphate buffered saline (PBS). After being blocked with PBS containing 5% goat serum and 30% bovine serum albumin (BSA) for 30 min at 4°C, cells were stained with cell-surface antibodies for 30 min at 4°C and washed twice with PBS containing 2.5% BSA. The Foxp3 Fixation and Permeabilization kit (eBioscience) was used according to the instructions of the manufacturer, and cells were incubated overnight in 1.6% paraformaldehyde (PFA) PBS with 100 nM iridium nucleic acid intercalator (Fluidigm) and lastly incubated with intracellular antibodies in permeabilization buffer for 30 min at 4 C after being washed twice with Foxp3 permeabilization buffer and twice with FACS buffer. Cells were washed, resuspended at a concentration of one million cells/ml with water containing EQ Four Element Calibration Beads (Fluidigm), and analyzed within 12 h on a Helios CyTOF System (Fluidigm) at an event rate of <500 events/s.

#### 2.3.2 Panel Design, CyTOF Data Acquisition, and Visualization

A list of 42 metal-tagged monoclonal antibodies including their label, clone, source, cat. number, dilution, and staining information was provided in **Supplementary Table S1**. All data were produced on a Helio3 CyTOF Mass Cytometer (Fluidigm) and normalized by the bead-based normalization software, which used the intensity values of a sliding window of these bead standards to correct for instrument fluctuations over time and among samples. The normalized FCS files were loaded into Cytobank (www.cytobank.org) to define CD45^+^ viable single living cells and other subpopulations. The gating strategies were shown in **Supplementary Figure S1**. Then FCS files were loaded into R and analyzed with an in-house written script. Cluster-colored tSNE maps and phenotype heatmaps were generated by the expression levels of markers with arcsin*h* transformation from 5,000 cells of each sample except Tregs and B cells (all cells). Hyperparameters were set as perplexity of 30, theta of 0.5, and iterations of 1,000 per 100,000 analyzed cells.

### 2.4 Statistical Analyses

RPLs were firstly stratified by embryonic chromosome: RPL-NK was composed of six RPLs with normal chromosome and RPL-AK was composed of nine RPLs with chromosome aberrations, while NC-NK was composed of five controls. One-way ANOVA analysis was applied followed by the Benjamini, Krieger, and Yekutieli test, and the adjusted false discovery rate (FDR) <0.05 was considered significant. Then, RPLs were compared with controls without stratification and Student’s *t*-test was used with *P*-value <0.05 considered as statistical significance. GraphPad Prism version 9 software was used for the statistical analyses.

## 3 Results

### 3.1 The General Immune Landscapes of RPL

**Figure 1:**
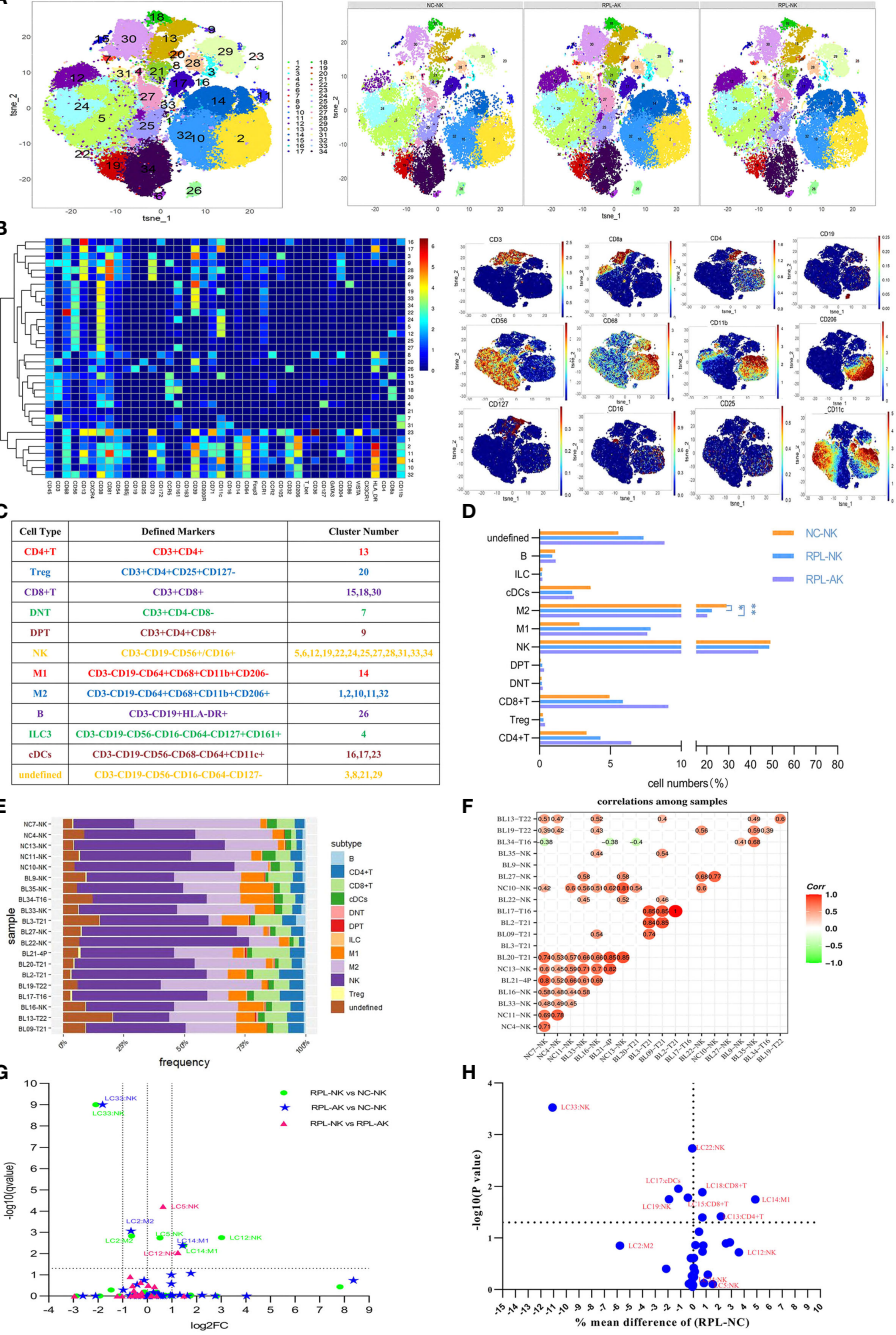
The general immune landscape of RPL. **(A)** CD45^+^ *t*-distributed stochastic neighbor embedding (tSNE) with all samples combined (left) and composite tSNE for groups (right). **(B)** Heatmap with clusters and the expression of 42 markers (left) and expression plots of the key markers (right). **(C)** Sum of cell subtypes, clusters, and their defined markers. **(D)** Graph showing cell numbers (%) in CD45^+^ cell population, * adjusted *P* < 0.05, ** adjusted *P* < 0.001. **(E)** Stack graph showing the frequency of subtypes of each sample. Samples were named by their code and embryonic chromosome result. **(F)** Pearson’s correlation coefficient used to evaluate the correlations among samples. **(G)** Volcano plot showing differential clusters when RPL was stratified. Fold change (FC) = the mean percent ratio of RPL-NK over NC-NK in RPL-NK vs. NC-NK, and so on. A dotted grid line *Y* = −log10 (0.05) = 1.30103 showing no statistical significance. Two dotted grid lines at *X* = 1, −1 showing increased two-fold or decreased half. **(H)** Volcano plot showing the differential clusters between RPL and controls when compared without stratification.

CD45^+^ leukocytes were clustered by phenograph (**Figures 1A–C** and **Supplementary Figure S1**). The percentages of M2 macrophages statistically decreased in both RPL subgroups when they were stratified, compared with controls (**Figure 1D**), while variation existed among the individuals (**Figures 1E, F**). The M1 macrophage LC14 (individually labeled) increased more than two-fold and the M2 cluster LC2 decreased statistically but less than half in each RPL subgroup. NK cluster LC33 decreased more than half in both RPL subgroups, while clusters LC5 and LC12 increased (no more than two-fold and more than two-fold, respectively) only in RPL with normal chromosome (**Figure 1G** and **Supplementary Table S2**). When RPLs were not stratified, NK cluster LC33 and M1 cluster LC14 remained statistically different (**Figure 1H**).

### 3.2 CD39^high^ NK Cells Decreased in RPL

**Figure 2:**
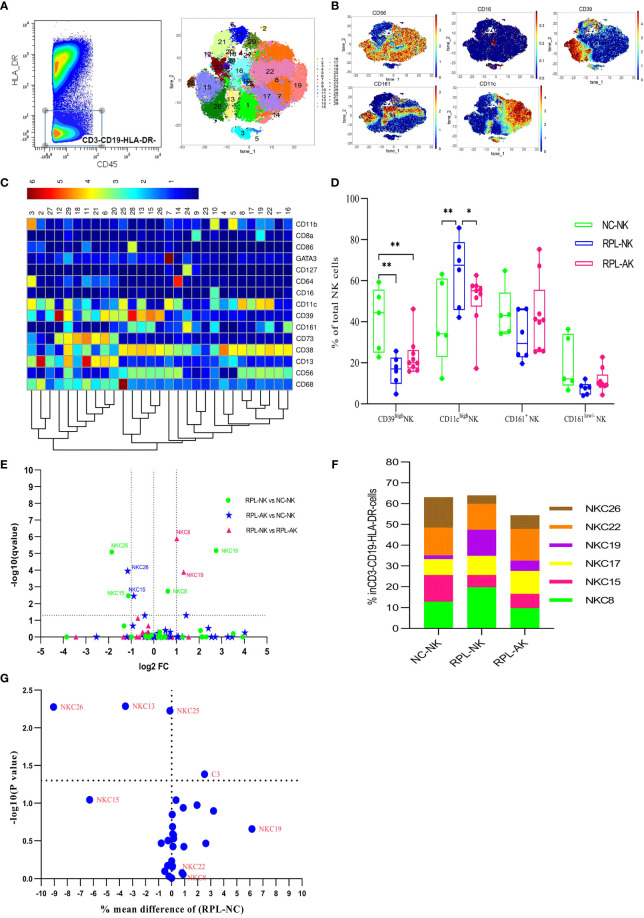
Cluster analysis of ILCs in the CD3^−^CD19^−^HLA-DR^−^ population. **(A)** CD3^−^CD19^−^HLA-DR^−^ population was manually gated (left) and tSNE in all samples combined (right). **(B)** Heatmap showing the expression profile of key markers. **(C)** Heatmap used for the cluster classification with the expression of markers in the CD45^+^CD3^−^CD19^−^HLA-DR^−^ population. **(D)** Box plot showing the comparison of CD39^high^ NK cells and CD11c^high^ NK cells. **(E)** Volcano plot showing the differential clusters when RPLs were stratified by embryonic chromosome. **(F)** Map with cumulative mean values for differential clusters and/or the most abundant three clusters. **(G)** Volcano plot showing the differential clusters when RPLs were not stratified. * adjusted *P* < 0.05, ** adjusted *P* < 0.001.

ILCs were clustered from manually gated CD45^+^CD3^−^CD19^−^HLA-DR^−^ subpopulation (**Figures 2A–C** and **Supplementary Figure S1**). The overall frequency of CD39^high^NK cells and two CD39^high^ NK cell subsets (NKC15, NKC26) decreased significantly in each RPL subgroup when RPLs were stratified, compared with controls (**Figures 2D–F** and **Supplementary Table S2**). If not stratified by embryonic chromosome, differences of NKC26, NKC13, and NKC25 between RPL and controls were obvious (**Figure 2G**). Only one NK ILC cluster (cluster 24) was identified and no difference was detected (data not shown). The phenotype signature of NKC26 was mirrored to LC33 (**Figures 8A, B**).

### 3.3 CCR2*^−^* CD11c^high^ Macrophages Increased, While Less Inflammatory CCR2*^−^* CD11c^low^ Decreased in RPL

**Figure 3:**
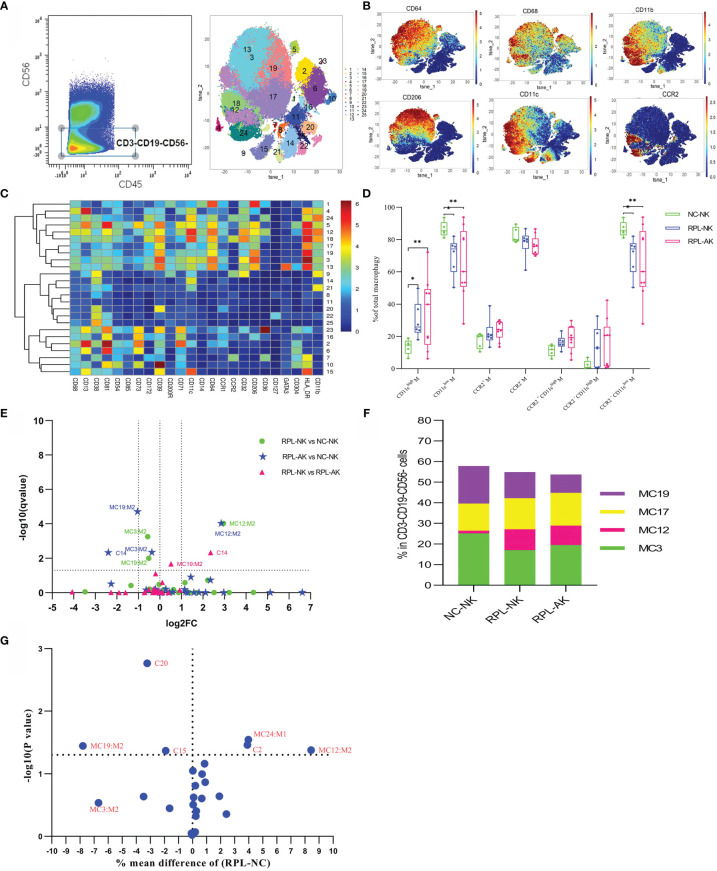
Cluster analysis of myeloid macrophages and DC in the CD3^−^CD19^−^CD56^−^ population. **(A)** Cells manually gated from the CD45^+^CD3^−^CD19^−^CD56^−^ population (left) and tSNE in all samples combined (right). **(B)** Marker expression profile of the key markers. **(C)** Heatmap with the expression of markers in the CD45^+^CD3^−^CD19^−^CD56^−^ population. **(D)** Box plot showing the comparison of total CD11c^high^, CD11c^low^, and/or CCR2^−^CD11c^low^ when RPLs were stratified. **(E)** Volcano plot showing the differential clusters when RPLs were stratified by embryonic chromosome. **(F)** Map with cumulative mean values for differential clusters and/or the most abundant three clusters. **(G)** Volcano plot showing the differential clusters when RPLs were not stratified. * adjusted *P* < 0.05, ** adjusted *P* < 0.001.

The CD45^+^CD3^−^CD19^−^CD56^−^ subpopulation was gated manually to analyze myeloid macrophages and DCs (**Supplementary Figure S1**). Macrophages were phenotyped based on the expression of CD64, CD68, and CD11b, while DCs were characterized by CD64^−^CD11c^+^ (**Figures 3A–C**). Compared with controls, total CD11c^high^ macrophages increased, while CD11c^low^ or CCR2^−^CD11c^low^ macrophages decreased with MC12 increased more than two-fold, but MC3 and MC19 decreased less than half in RPL subgroups (**Figures 3D–F** and **Supplementary Table S2**). MC19 and MC12 were still obviously different when RPLs were not stratified (**Figure 3G**). Six DC clusters were identified with no significant difference noticed in RPL (data not shown). The phenotype signature of MC3 was mirrored to LC2 (**Figures 8A, C**).

### 3.4 CD38^high^HLA-DR^high^CCR5^high^CD8^+^ T and CD8^+^CD56^+^ NKT Cells Decreased in RPL

**Figure 4:**
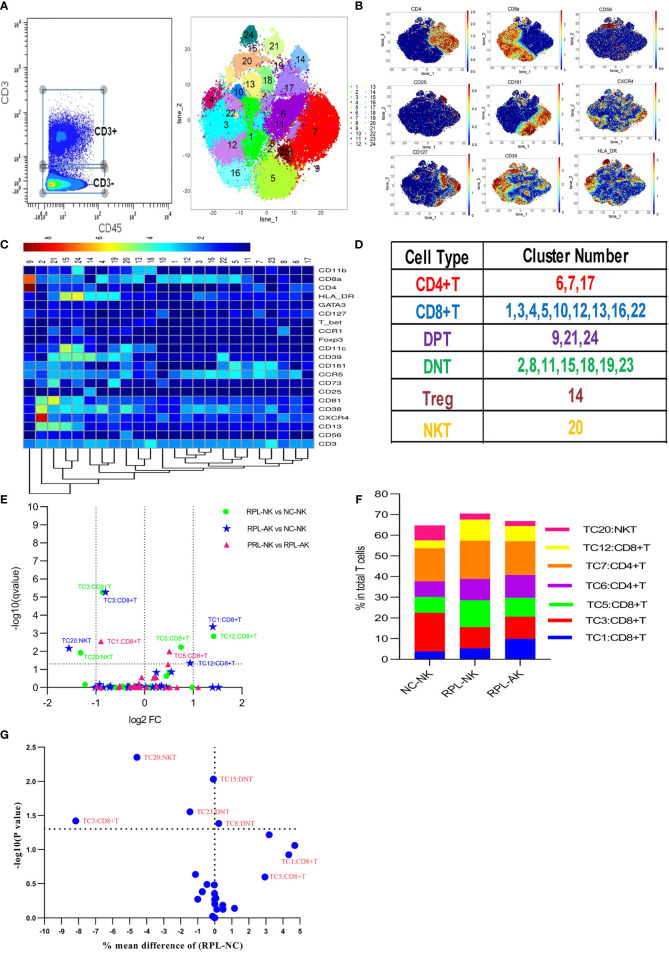
CD3^+^ T-cell cluster analysis. **(A)** CD3^+^ T cells manually gated on CD45^+^ leukocytes by CD3 expression (left) and CD3^+^ T-cell tSNE in all samples combined (right). **(B)** Expression profile of the key markers. **(C)** Heatmap with clusters and the expression of markers in CD3^+^ T cell. **(D)** Sum of the clusters for each subset. **(E)** Volcano plot showing the differential clusters when RPLs were stratified by embryonic chromosome. **(F)** Map with cumulative mean values for differential clusters and/or the most abundant three clusters. **(G)** Volcano plot showing the differential clusters when not stratified.

CD3^+^ T cells were firstly clustered (**Figures 4A–D**). The CD8^+^ T-cell cluster TC3, characterized by CD38^high^HLA-DR^high^CCR5^high^, decreased nearly by half, while CD8^+^CD56^+^ NKT cells (TC20) reduced more than half in each RPL subgroup, compared with controls (**Figures 4E, F**). Except TC3 and TC20, three CD4^−^CD8^−^ T (DNT) subsets were significantly different between RPL and controls if RPLs were compared without stratification (**Figure 4G**).

### 3.5 CD38^high^HLA-DR^high^CD161^high^ Teff Decreased in RPL

**Figure 5:**
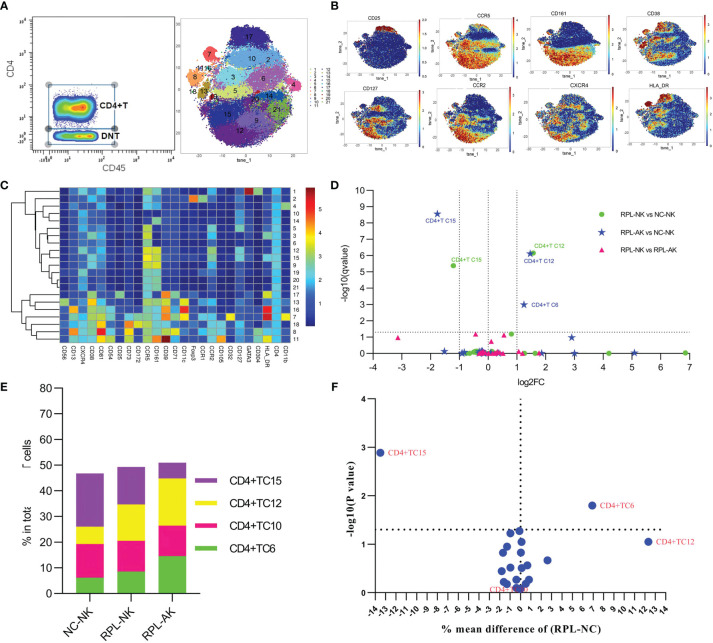
In-depth analysis of CD4^+^ T cells. **(A)** CD4^+^ T cells manually gated on concatenated CD45^+^CD3^+^CD8^−^ population (left) and CD4^+^ T-cell tSNE in all samples combined (right). **(B)** Expression profile of the key markers. **(C)** Heatmap with clusters and the expression of markers in CD4^+^ T-cell clusters. **(D)** Volcano plot showing the differential clusters when RPLs were stratified by embryonic chromosome. **(E)** Map with cumulative mean values for differential clusters and/or the most abundant three clusters. **(F)** Volcano plot showing the differential clusters when RPLs were not stratified.

To better phenotype CD4^+^ T cells, they were clustered from gated CD8^−^ T cells (**Figures 5A–C** and **Supplementary Figure S1**). CD4^+^CCR2^+^CCR5^+^ defined an effector T-cell (Teff) phenotype. CD38^high^HLA-DR^high^CD161^high^ Teff subset (CD4^+^TC15) decreased more than half, while CXCR4^+^CD161^high^ Teffs (CD4^+^TC12) increased more than two-fold in each RPL subgroup, compared with controls (**Figures 5D, E** and **Supplementary Table S2**). However, the difference of CXCR4^+^CD161^high^ Teffs was obvious only when RPLs were stratified (**Figure 5F**).

### 3.6 CD39*^−^* CCR5^high^ Tregs Might Be Enhanced in RPL

**Figure 6:**
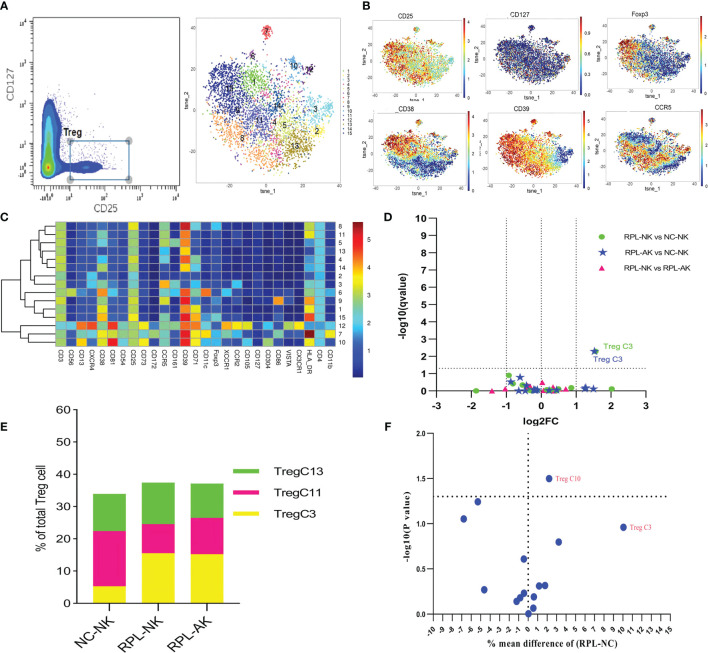
In-depth analysis of Tregs. **(A)** Tregs manually gated on concatenated CD45^+^CD25^+^CD127^−^ population (left) and Tregs tSNE in all samples combined (right). **(B)** Expression profile of the key markers. **(C)** Heatmap with clusters and the expression of markers in Tregs. **(D)** Volcano plot showing the differential clusters when RPLs were stratified by embryonic chromosome. **(E)** Map with cumulative mean values for differential clusters and/or the most abundant three clusters. **(F)** Volcano plot showing differential clusters when RPLs were compared without stratification.

Tregs were gated as CD4^+^CD25^+^CD127^−^ and clustered automatically (**Figures 6A–C** and **Supplementary Figure S1**). The most abundant cluster TregC3, characterized by CD39^−^CCR5^high^, increased more than two-fold in each RPL subgroup (**Figures 6D, E** and **Supplementary Table S2**). While this difference was not significant between RPL and controls if they were not stratified (**Figure 6F**).

### 3.7 CD85^high^CD39^high^CD73*^−^* B Cells Might Decrease in RPL

**Figure 7:**
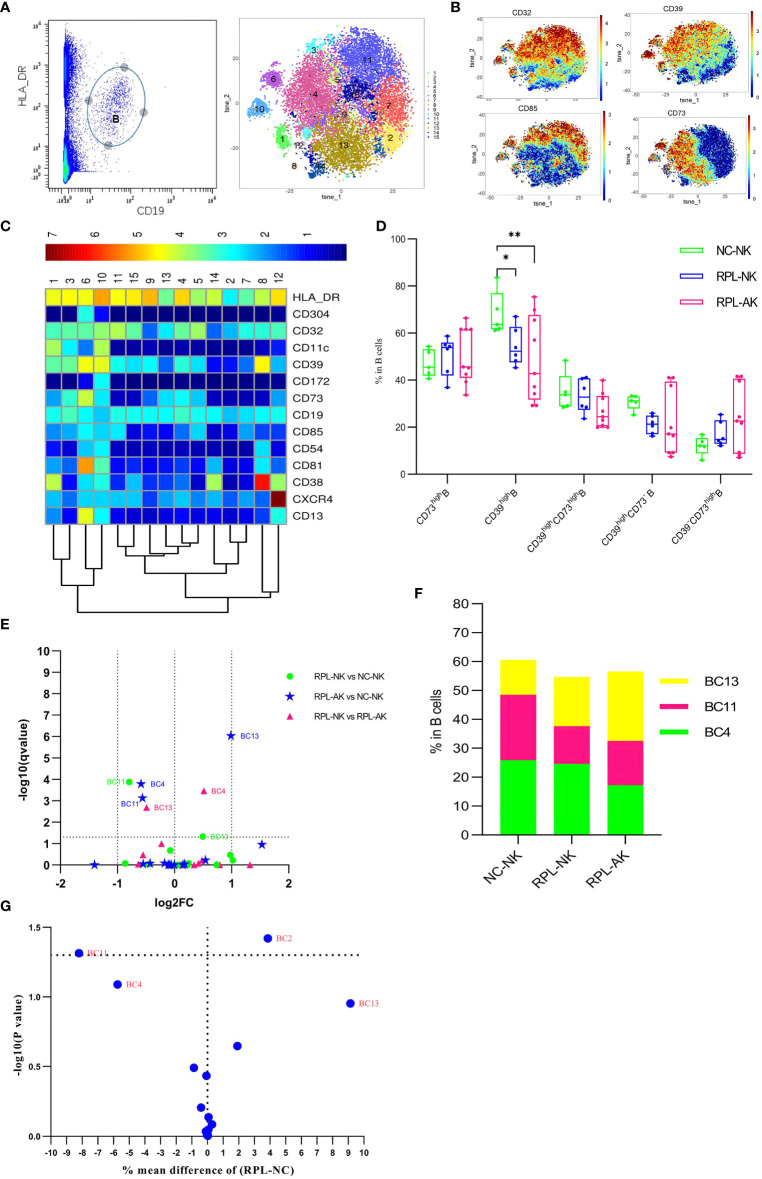
B-cell cluster analysis. **(A)** B cell manually gated on concatenated CD45^+^CD3^−^ population (left) and B-cell tSNE in all samples combined (right). **(B)** Expression profile of key markers. **(C)** Heatmap with clusters and the expression of markers in CD45^+^CD3^−^CD19^+^HLA-DR^+^ population. **(D)** Box plot showing the comparison of total CD39^high^ Breg cell when RPLs were stratified by embryonic chromosome. **(E)** Volcano plot showing the differential clusters when RPLs were stratified. **(F)** Map with cumulative mean values for differential clusters and/or the most abundant three clusters. **(G)** Volcano plot showing differential clusters when RPLs were compared as an entirety. * adjusted *P* < 0.05, ** adjusted *P* < 0.001.

As subsets of B cells could mediate immune tolerance, CD19^+^HLA-DR^+^ B cells were clustered (**Figures 7A–C**). The overall frequency of CD39^high^ B cells decreased in RPL subgroups, compared with controls (**Figure 7D**). Cluster BC11, characterized by CD85^high^CD39^high^CD73^−^, decreased statistically though less than half, while cluster BC13, characterized by CD39^low^CD73^high^, increased much more in RPL with embryonic chromosomal aberrations (**Figures 7E, F** and **Supplementary Table S2**). When RPLs were compared as an entirety, only the CD85^high^CD39^high^CD73^−^ B subset was statistically significant (**Figure 7G**).

### 3.8 CD11c^high^ NK and CD161^high^CD8^+^ T Cells Were Enriched Only in RPL With Normal Embryonic Chromosome

The overall frequency of CD11c^high^ NK cells (**Figure 2D**) and two CD11c^high^ NK subsets enriched significantly with NKC8 increased less than and NKC19 more than two-fold enriched in RPL with normal embryonic chromosome (**Figures 2C, D** and **Supplementary Table S2**).

**Figure 8:**
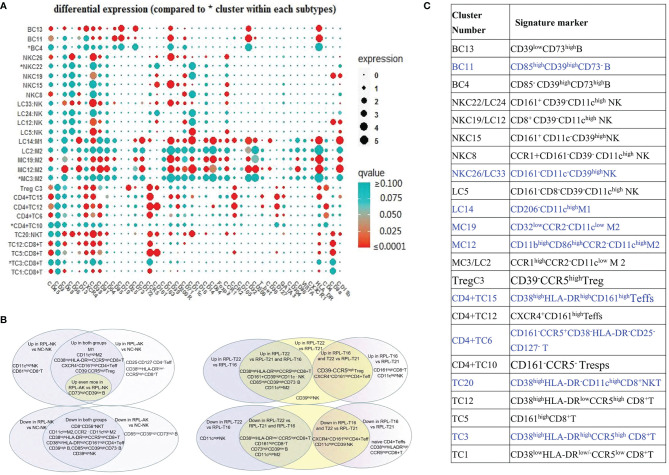
Sum of differential clusters. **(A)** Bubble map of all markers, compared with the most abundant cluster indicated by *. Circle size showing the expression level (arcsin*h*) and its colors showing *P*-value [range from <0.0001 (red) to >0.100 (green)]. **(B)** Sum of the characteristics of differential clusters and clusters with blue font showing they were differential whenever RPLs were stratified or not. **(C)** Venn diagram summarizing the differential clusters when RPLs were stratified by embryonic chromosome (left) or the differences among three types of fetal aneuploidy (right).

CD161^high^CD8^+^ T cells (TC5) also increased statistically but less than two-fold, which might indicate local inflammatory activation (**Figures 4A–E** and **Supplementary Table S2**). The phenotype signature of NKC19 and NKC22 was mirrored to LC12 and LC24, respectively (**Figures 8A, C**).

### 3.9 Naive CD8^+^ T/CD4^+^ T Increased Only in RPL With Embryonic Chromosomal Aberrations

The inactivated or naive CD38^low^HLA-DR^low/−^CCR5^low^CD8^+^ T cells (TC1) enriched more than two-fold in RPL with embryonic chromosomal aberrations, compared with controls or RPL without chromosomal aberrations (**Figure 4E** and **Supplementary Table S2**). A similar tendency was observed in the CD38^−^HLA-DR^−^CD25^−^CD127^−^CD4^+^ T subset (CD4^+^ TC6) and its differential remained obviously when RPLs were not stratified (**Figures 5D–F** and **Supplementary Table S2**). B-cell cluster BC4, characterized by CD85^−^CD39^high^CD73^high^, decreased significantly RPL with chromosomalaberrations, but the difference was not obvious if RPLs were compared without stratification (**Figures 7C–E** and **Supplementary Table S2**).

### 3.10 The Immune Atlas Among RPLs With Embryonic Aneuploid Is Heterogeneous

**Figure 9:**
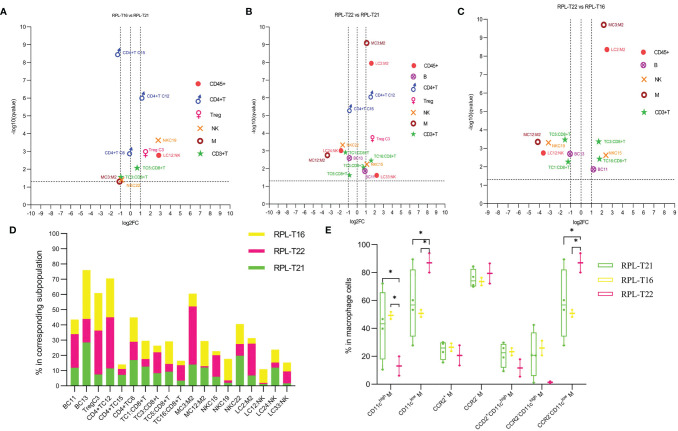
Immune atlas of RPL with embryonic aneuploidy. **(A)** Volcano plot showing differential clusters in RPL with embryo T16 (RPL-T16), compared with RPL with T21 (RPL-T21). log2FC >1 indicating this cluster increased more than two-fold in RPL-T16 compared with RPL-T21, and so on. **(B)** Volcano plot showing differential clusters in RPL with T22 (RPL-T22), compared with RPL-T21. **(C)** Volcano plot showing differential clusters in RPL-T16, compared with RPL-T22. **(D)** Map with cumulative mean values for differential clusters of RPL with embryos T21, T22, and T16. **(E)** Frequencies of macrophage subsets in each aneuploidy. * adjusted *P* < 0.05.

Compared with RPL with embryo T21, pro-inflammatory subsets including CD8^+^CD39^−^CD11c^high^ NK, CXCR4^+^CD161^high^ Teffs, and CD39^−^CCR5^high^ Tregs increased more than two-fold, while suppressive or less pro-inflammatory subsets decreased nearly half in RPL with T16 (**Figures 8B** **,** **9A, D** and **Supplementary Table S3**). Some suppressive or pro-inflammatory subsets increased more than two-fold, while part of the pro-inflammatory subsets decreased more than half in RPL with T22 (**Figures 9B, D** and **Supplementary Table S3**). Suppressive subsets increased, while pro-inflammatory decreased in RPL with T22, when compared with RPL with T16 (**Figures 8B** **,** **9C, D** and **Supplementary Table S3**). The total CD11c^high^ macrophages decreased, while CD11c^low^ or CCR2-CD11c^low^ macrophages increased in RPL with T16 and T22 compared with RPL-T21 (**Figure 9E**). These results should be assessed with more participants.

## 4 Discussion

Previous attention has been mainly focused on the immune mechanism of RPL with normal embryonic chromosome. In the present study, stratification analysis showed that the immune status of RPL with chromosomal aberrations was pro-inflammatory, as suppressive CD39^high^dNK1 and CCR2^−^CD11c^low^ macrophages decreased, while pro-inflammatory CCR2^−^CD11c^high^ and CD206^+^M2 subset increased, which were found in RPL without chromosomal aberrations in previous studies and in our study. Secondly, we observed that the fully activated CD8^+^ T/CD161^high^CD4^+^ Teff cells and CD8^+^CD56^+^ NKT cells decreased in RPL whether stratified or not. Moreover, the inactivated and naive CD8^+^/CD4^+^ T cells were enriched statistically only in RPL with chromosomal aberrations, while CD11c^high^ NK and CD161^high^CD8^+^ T cells increased only in RPL without aberrations.

By far, five decidual NK-cell subsets are identified and only dNK1 cells express CD39 (20, 25). CD39^high^dNK1 cells, confined to late luteal phase and pregnant uterus, mediate immune tolerance and vascularization by producing galectin-1 and galectin-9 (25). Their frequency decreases significantly in RPL with normal embryonic chromosome (20), and we detect similar results both in RPL with and without chromosomal aberrations The above research also indicates RPL women have obviously increased frequency of dNK3 (20). dNK3 may be mirrored to CD11c^high^ NK cells because they are both pro-inflammatory and negative for CD39 expression (25). However, CD11c^high^ NK cells increase only in RPL without chromosomal aberrations by stratification analysis. These data support the fact that decreased CD39^high^dNK1 cells are involved in RPL occurrence, while whether NK cells from RPL with normal chromosome have a defective differentiation process deserve further discussion because dNK cells differentiate with sequential expression of killer cell immunoglobulin-like receptors (KIRs) and CD39 but lose CD11c expression eventually (25).

M1/M2 macrophage unbalance is correlated with spontaneous abortion (26, 27), and our results in RPL with and without chromosomal aberrations are consistent with previous findings. It should be noted that one of the CD206^+^ M2 subsets, the high expression of CD11c, increased remarkably. M2 macrophages expressing pro-inflammatory signatures have been reported from unexplained RPL decidua by scRNA-seq (22) and aorta–gonad–mesonephros (AGM) macrophages by CyTOF (28). It has been suggested that decidual macrophages can be classified into CD11c^high^ and CD11c^low^ subsets or three subsets combined with CCR2 expression, of which the CCR2^−^CD11c^high^ macrophages have the highest phagocytic capacity, while CCR2^−^CD11c^low^ exhibit the fewest inflammatory properties (29). Our investigations support this proposal and prove that macrophages in RPL decidua are preferentially pro-inflammatory.

Decidual CD8^+^CD56^+^ NKT cells are firstly described in a recent scRNA-seq paper (23), and they decreased significantly in our investigation whether RPLs were stratified or not. They might be immune suppressive (30), but this needs to be confirmed further (31). CD161^high^CD8^+^ T cells display a pro-inflammatory Tc1/Tc17 phenotype (32, 33), and more than 90% of them are mucosal-associated invariant T cells (MAIT) (34, 35). The roles of MAIT cells in pregnancy are evidenced in RPL peripheral blood (20, 36), while the association between decidual CD161^high^CD8^+^ T cells and RPL without chromosomal aberrations is firstly revealed. Except for the Th1/Th17 phenotype (37), CD161^high^CD4^+^ T cells can exert an inhibiting effect depending on their developed microenvironment (38). Dual expressions of CD38 and HLA-DR indicate the full activation of T cells and immune suppression (39–41); thus, CXCR4^+^CD161^high^CD4^+^ T cells might be pro-inflammatory, whereas fully activated CD161^high^CD4^+^ T cells are suppressive. Both the fully activated CD38^high^HLA-DR^high^CCR5^high^CD8^+^ T and CD161^high^CD4^+^ T cells are firstly found to be decreased statistically in RPL regardless of their stratification. In a word, regulatory or suppressive CD4^+^ T/CD8^+^ T subsets play an important role in maintaining pregnancy.

The suppressive function of Tregs is governed by CD39 (42, 43), but CCR5^high^ Tregs have pro-inflammatory potential (44); thus, CD39^−^CCR5^high^ Tregs are pro-inflammatory. CD39^high^ B cells are mainly Bregs and they possess inhibiting capacity through the adenosine pathway or secreting IL-10 (45, 46). Herein, the stratification analysis showed that CD39^−^CCR5^high^ Tregs increased, while CD85^high^CD39^high^CD73^−^ Bregs decreased both in RPL with and without aberrations. They highlight the key roles of CD39^high^ Tregs/Bregs in successful pregnancy. However, when RPLs are not stratified, their differences are not obvious because of significant individual variations.

Stratification analysis by embryonic chromosome is very necessary. We notice that the immune atlas of RPL with chromosomal aberrations is similar in multiple cells to that of RPL without aberrations. Meanwhile, their CD32^low^CCR2^−^CD11c^low^ M2 and CD39^low^CD73^high^ B cells decrease or increase much more than RPL without aberrations, whereas the inactivated and naive CD8^+^/CD4^+^ T cells were enriched only in them. Whether they are associated with chromosomal alterations deserves investigating in the future. In addition, the only increased frequency of CD11c^high^ NK and CD161^high^CD8^+^ T cells in RPL with normal chromosome reminds us that their immune atlas might be more pro-inflammatory than RPL with aberrations.

When RPLs are not stratified, almost only differential clusters with cutoff of log2FC more than 1 both in RPL with and without chromosomal aberrations remain statistically significant. Of course, many clusters with no statistical difference during stratification analysis are of significance, whereas CXCR4^+^CD161^high^ Teffs and CD39^−^CCR5^high^ Tregs are no longer different though they increase more than two-fold in both. These are related with individual variations and cell counts of each cluster, and thus, it may lead to poor identification of differential clusters when RPLs were not stratified or the cutoff is more than 1. They can be proven by further immunohistochemical method or flow cytometry.

Variations among RPLs with different embryo aneuploids shall be analyzed. Decidual immune compartments of RPL with embryo T16 have been reported to be no different from those of women undergoing selective terminations (47). One limitation of this study is that only six antibodies (CD45, CD3, CD56, CD68, CD69, and CD25) have been used for immunohistological staining. Differences have been noticed in our investigation and these need further validation due to their limited participants.

In summary, we provide a comprehensive immune atlas of RPL decidual by CyTOF and discover some variations similar to the findings by scRNA-seq or flow cytometry. More importantly, we detect that immunological abnormalities of RPL with embryonic chromosomal aberrations are pro-inflammatory too, but some differences exist between RPL with and without aberrations.

## Data Availability Statement

The original contributions presented in the study are included in the article/**Supplementary Material**. Further inquiries can be directed to the corresponding author.

## Ethics Statement

The studies involving human participants were reviewed and approved by the Ethics Committee of Women’s Hospital, Zhejiang University School of Medicine (approval number IRB-20200126-R). The patients/participants provided their written informed consent to participate in this study. Written informed consent was obtained from the individual(s) for the publication of any potentially identifiable images or data included in this article.

## Author Contributions

ZW and MD conceived and designed the project. ZW, YX, MW, and XJ collected the samples and consent forms. ZW and GL analyzed the data. JQ and MD gave their constructive suggestions during data analysis. ZW wrote the draft. ZW and MD edited the manuscript. All authors contributed to the article and approved the submitted version.

## Funding

This research was supported by the Natural Science Foundation of China (No. 81370726), Key Research and Development Plan of Zhejiang Province (No. 2019C03025), Natural Science Foundation of Zhejiang Province (No. LQ19H040011), Program for Education Department of Zhejiang Province (No. Y201941996), and Zhejiang Province Medical and Health Technology Project (Nos. 2019KY431 and 2020KY616).

## Conflict of Interest

The authors declare that the research was conducted in the absence of any commercial or financial relationships that could be construed as a potential conflict of interest.

## Publisher’s Note

All claims expressed in this article are solely those of the authors and do not necessarily represent those of their affiliated organizations, or those of the publisher, the editors and the reviewers. Any product that may be evaluated in this article, or claim that may be made by its manufacturer, is not guaranteed or endorsed by the publisher.
