# Supplementary material for: Pro-Inflammatory Signature in Decidua of Recurrent Pregnancy Loss Regardless of Embryonic Chromosomal Abnormalities
Source: Front Immunol. 2021 Dec 8;12:772729. doi: 10.3389/fimmu.2021.772729 (PMC8694032; doi:10.3389/fimmu.2021.772729)
Supplement: Supplementary file 1 [file DataSheet_1.pdf]

Supplemental Materials

Table S1. list of markers used in the present CyTOF panel including their label,clone,source,cat,dilution and staining information.

List	Label	Marker	clone	Source	Cat	Dilution	Staining
1	89Y	CD45	HI30	BioLegend	304002	100	Surface
2	115In	CD3	UCHT1	BioXcell	BE0231	200	Surface
3	139La	CD68	Y1/82A	BioLegend	333802	100	Intracellular
4	141Pr	CD56	NCAM16.2	BD	559043	800	Surface
5	142Nd	CD13	WM15	Biolegend	301702	50	Surface
6	143Nd	CXCR4	12G5	Biolegend	306502	100	Surface
7	144Nd	CD38	HIT2	BioLegend	303502	100	Surface
8	145Nd	CD81	5A6	BioLegend	349502	50	Surface
9	146Nd	CD54	HA58	BioLegend	353102	100	Surface
10	147Sm	CD85j	GHI/75	BioLegend	CD85j	200	Surface
11	148Nd	CD19	HIB19	BioLegend	333802	200	Surface
12	149Sm	CD25	24212	RD	MAB1020	400	Surface
13	150Nd	CD73	AD2	BioLegend	344002	100	Surface
14	151Eu	CD172a	SE5A5	BioLegend	323802	200	Surface
15	152Sm	CCR5	J418F1	BioLegend	350002	400	Surface
16	153Eu	CD161	HP-3G10	BioLegend	339902	100	Surface
17	154Sm	CD163	GHI/61	RD	MAB16072-100	100	Surface
18	155Gd	CD39	A1	BioLegend	328202	100	Surface
19	156Gd	CD200R	OX-108	BioLegend	329302	100	Surface
20	157Gd	CD71	GY1G4	BioLegend	334102	100	Surface
21	158Gd	CD11c	BU15	BioLegend	337202	400	Surface
22	159Tb	CD16	3G8	BioLegend	302014	100	Surface
23	160Gd	CD14	M5E2	BioLegend	301810	50	Surface
24	161Dy	CD64	10.1	BioLegend	305002	50	Surface
25	162Dy	Foxp3	PCH101	eBioscience	14-4776-82	50	Intracellular
26	163Dy	CCR1	5F10b29	BioLegend	362902	50	Surface
27	164Dy	CCR2	K036C2	BioLegend	357202	100	Surface
28	165Ho	CD105	43A3	BioLegend	323214	100	Surface
29	166Er	CD32	FUN-2	BioLegend	303202	200	Surface
30	167Er	CD206	‘15-2	BioLegend	321127	100	Surface
31	168Er	Tbet	4B10	BioLegend	644825	100	Intracellular

32	169Tm	CD36	5-271	BioLegend	336202	400	Surface
33	170Er	CD127	A019D5	BioLegend	351302	100	Surface
34	171Yb	GATA3	TWAJ	eBioscience	14-9966-82	50	Intracellular
35	172Yb	CD304	12C2	BioLegend	354502	100	Surface
36	173Yb	CD86	Fun-1	RD	MAB141-100	100	Surface
37	174Yb	VISTA	#730804	RD	730804	50	Surface
38	175Lu	CX3CR1	K0124E1	BioLegend	355702	200	Surface
39	176Yb	HLA-DR	L243	BioLegend	307612	100	Surface
40	197Au	CD4	RPA-T4	BioLegend	300516	400	Surface
41	198Pt	CD8a	RPA-T8	BioLegend	301018	400	Surface
42	209Bi	CD11b	M1/70	BioLegend	101202	800	Surface

Table S2. Summarize of the different abundant clusters from RPL as compared to control （NC-NK）.

	NC-NK															
Differences occurred regardless of embryonic chromosomal aberrations																
	LC2	LC14	LC33	TC3	TC12	TC20	CD4+TC12	CD4+TC15	TregC3	BC11	BC13	MC3	MC12	MC19	NKC15	NKC26
RPL-NK	↓	↑	↓	↓	↑	↓	↑	↓	↑	↓	↑	↓	↑	↓	↓	↓
RPL-AK	↓	↑	↓	↓	↑	↓	↑	↓	↑	↓	↑	↓	↑	↓	↓	↓
Differences occurred only in RPL patients without embryonic chromosomal aberrations																
	LC5	LC12		TC5											NKC8	NKC19
RPL-NK	↑	↑		↑											↑	↑
Differences occurred only in RPL patients with embryonic chromosomal aberrations																
				TC1			CD4+TC6			BC4						
RPL-AK				↑			↑			↓						

Table S3. Summarize of the different abundant clusters from RPL-T16 and T21 as compared to RPL with embryo T22.

RPL-T22

Differences occurred both in RPL patients with embryo T21 and T16

	LC2	TC1	TC5	TC3	TC16	BC11	BC13	MC3	MC12	NKC15
RPL-T21	↓	↑	↑	↓	↓	↓	↑	↓	↑	↓
RPL-T16	↓	↑	↑	↓	↓	↓	↑	↓	↑	↓

Differences occurred only in RPL patients with embryo T21

	LC24	LC33	CD4+TC15	CD4+TC12	TregC3	NKC22
RPL-T21	↑	↓	↑	↓	↓	↑

Differences occurred only in RPL patients with embryo T161

	LC12	NKC19
RPL-T16	↑	↑

Supplemental Figures and Legends

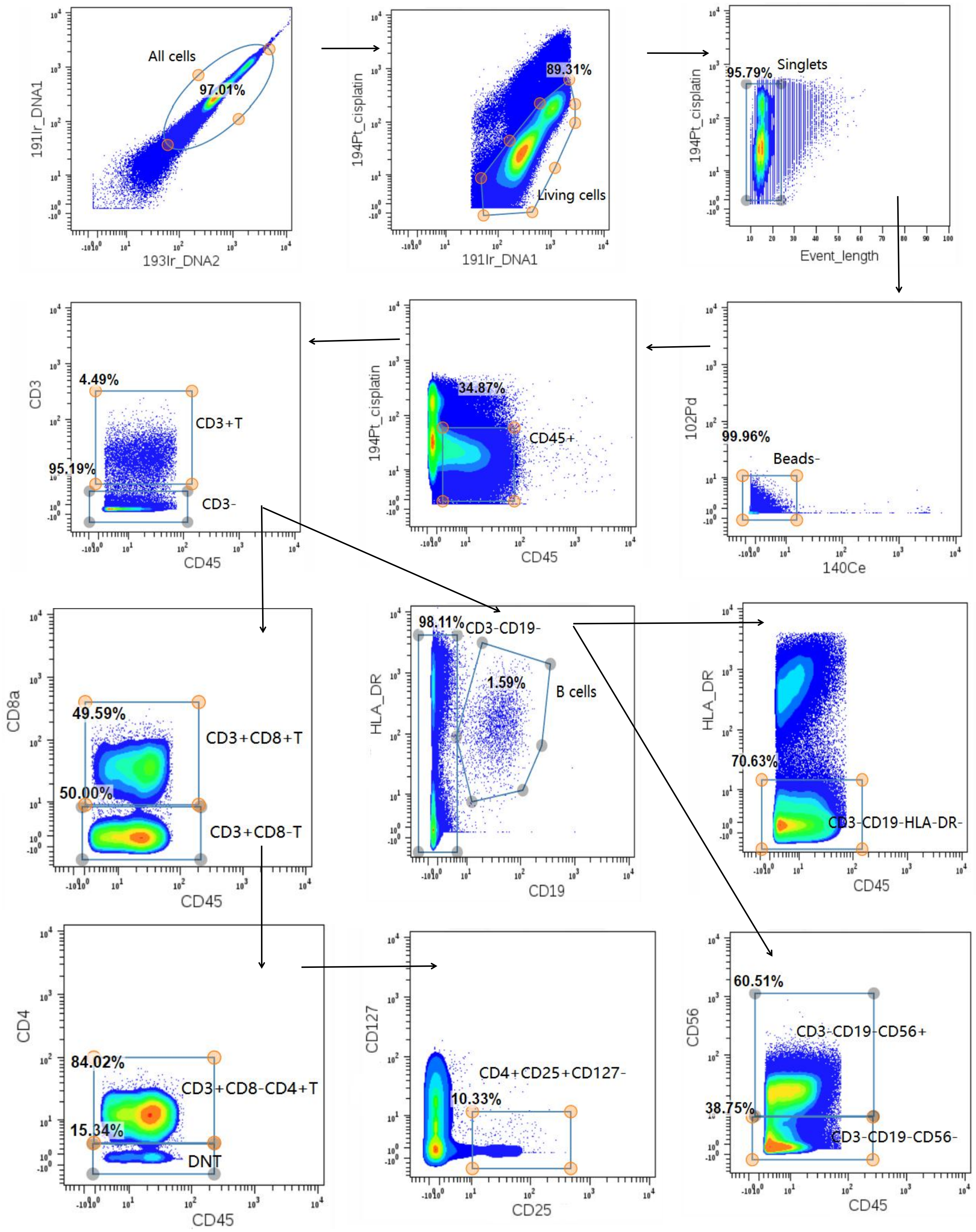

Figure S1. Gating strategy for Live CD45$^+$ leucocytes and different populations analyzed in present study. Iridium DNA double positive events were gated as all cells.Cisplatin DNA intercalator negative cells were gated as live cells and further for singlets by event-length, normalisation beads were then removed,at last they were separated by the expression of CD45. CD45$^+$ populations were further gated by CD3 expression. CD45$^+$CD3$^+$ could be identified as T cells and further used to gate CD8$^+$T by CD8a expression,CD4$^+$T cells were gated from CD3$^+$CD8$^-$T cells by CD4 expression and Treg cells were lastly gated from CD4$^+$T cell by CD25 and CD127 expression.CD45$^+$CD3$^-$ cells were firstly used to gate B cells by CD19 and HLA-DR expression while the CD3$^-$CD19$^-$ cells were used to gate CD3$^-$CD19$^-$HLA-DR$^-$ population by HLA-DR expression for analyzing ILCs and CD3$^-$CD19$^-$CD56$^-$ population by CD56 expression for analyzing myeloid macrophages and DCs.
